# Comparison of bloodstream and non-bloodstream infections caused by carbapenem-resistant *Klebsiella pneumoniae* in the intensive care unit: a 9-year respective study

**DOI:** 10.3389/fmed.2023.1230721

**Published:** 2023-09-14

**Authors:** Xiangyuan Sun, Xiaocui Zou, Boting Zhou, Tao Yin, Ping Wang

**Affiliations:** ^1^Department of Pharmacy, Xiangya Hospital, Central South University, Changsha Hunan, China; ^2^Department of Pharmacy, Lixian People’s Hospital, Lixian, Hunan, China; ^3^National Clinical Research Center for Geriatric Disorders, Xiangya Hospital, Central South University, Changsha, Hunan, China

**Keywords:** Carbapenem resistance, *Klebsiella pneumoniae*, bloodstream infection, non-bloodstream infection, intensive care unit

## Abstract

**Background:**

Bloodstream infections (BSIs) caused by carbapenem-resistant *Klebsiella pneumoniae* (CRKP) have received much attention. However, few studies have identified risk factors for CRKP BSIs in comparison to CRKP non-bloodstream infections (non-BSIs). This study aimed to compare the epidemiology, risk factors, and outcomes of CRKP BSIs and CRKP non-BSIs.

**Methods:**

We conducted a retrospective study of patients infected with CRKP in the ICU from January 2012 to December 2020. Clinical characteristics and outcomes were compared between CRKP BSIs and CRKP non-BSIs. Predictors associated with 28-day all-cause mortality in CRKP-infected patients were also evaluated.

**Results:**

326 patients infected with CRKP were enrolled, including 96 patients with CRKP BSIs and 230 with CRKP non-BSIs. The rates of CRKP BSIs in CRKP infections were generally raised from 2012 (12.50%) to 2020 (45.76%). Multivariate logistic analysis indicated that the use of carbapenems within the prior 90 days was an independent risk factor for CRKP BSIs (*p* = 0.019). Compared to CRKP non-BSIs, CRKP isolates in the CRKP BSI group were found to be non-susceptible to more tested carbapenems (*p* = 0.001). Moreover, the CRKP BSI group exhibited a higher mortality rate (*p* = 0.036). The non-susceptibility of CRKP isolates to more tested carbapenems (*p* = 0.025), a high SOFA score (*p* = 0.000), and the use of antifungal drugs within the prior 90 days (*p* = 0.018) were significant factors for 28-day all-cause mortality in CRKP-infected patients.

**Conclusion:**

The proportion of CRKP BSI increased progressively in CRKP-infected patients over 9 years. The use of carbapenems within the prior 90 days was an independent risk factor for the development of CRKP BSIs. The non-susceptibility of CRKP isolates to more tested carbapenems and a higher mortality rate were found in the CRKP BSI group.

## Introduction

*Klebsiella pneumoniae* (KP) is a gram-negative pathogen commonly found in healthcare facilities, and the rise of carbapenem-resistant *Klebsiella pneumoniae* (CRKP) has become a global challenge (1, 2). The rates of CRKP have significantly increased in both China and other countries (3–5). According to reports from the Chinese drug-resistant bacteria surveillance system, the resistance rates of *K. pneumoniae* to imipenem and meropenem increased from 3% and 2.9% in 2005 to 25% and 26.3% in 2018 (6). This alarming increase in CRKP resistance poses a serious threat to human health. Studies have shown that CRKP is often associated with high mortality and morbidity, especially for critically ill patients in the intensive care unit (ICU) (7–10).

CRKP is mainly associated with various types of hospital-acquired infections (11), such as bloodstream infections (BSIs), respiratory and urinary tract infections, skin and soft tissue infections (SSTIs). Notably, CRKP bloodstream infections are the most commonly reported (12). BSI was associated with increased mortality (13), and CRKP BSI was also associated with high mortality (14). Previous studies have primarily focused on comparing the risk factors of CRKP BSI with those of CSKP BSI (15–17) or non-CRKP BSI (18). These studies reported risk factors for KP resistance to carbapenems in patients with KP BSI or risk factors for CRKP infection in patients with BSI. However, the identified risk factors may not accurately reflect the risk of developing CRKP BSIs in patients with CRKP infections.

Although no studies have compared BSIs and non-BSIs caused by CRKP, there have been reports on other pathogens. For example, Su et al. (19) compared the clinical characteristics of *Acinetobacter* spp. BSIs and non-BSIs. In addition, studies have been conducted comparing bacteremic and non-bacteremic pneumonia caused by *S. pneumoniae* and *Acinetobacter baumannii* (20, 21), as well as bacteremic and non-bacteremic acute pyelonephritis due to *Escherichia coli* (22). Given the rapid progression and high motility of CRKP, it is still crucial to identify the risk factors for CRKP BSIs in patients in infected patients.

In this study, we aimed to compare the clinical characteristics of BSIs and non-BSIs caused by CRKP, including epidemiology, risk factors for developing CRKP BSIs, and outcomes. Additionally, we evaluated the risk factors for 28-day mortality in patients infected with CRKP.

## Methods

### Study design and patients

A case-control study was conducted in the intensive care unit (ICU) of Xiangya Hospital, a 3,600-bed teaching hospital in Changsha, China. Patients diagnosed with CRKP infection and who performed blood cultures during hospitalization in the ICU of Xiangya Hospital between January 2012 and December 2020 were collected. Patients were excluded if they had been infected with CRKP prior to admission, colonized with CRKP isolates, or were infected with polymicrobial BSIs. If the patients had multiple positive blood cultures, we only recorded the first positive result. Positive culture of CRKP in other sites were also like this. In our study, patients with CRKP infections were divided into two groups according to the diagnosis of CRKP BSI: patients with CRKP BSIs were in the case group, and patients with CRKP infections in other sites (non-BSIs) were in the control group. The study was approved by the Ethics Committee of Xiangya Hospital Central South University (2018091076).

### Data collection

Patient and CRKP specimen information was collected from electronic medical records. The variables included age, sex, admission and discharge dates, APACHE II and SOFA scores at admission, comorbidities (hypertension, diabetes, coronary artery disease, hepatitis/cirrhosis, malignancy, cerebrovascular disease), previous admission (community, or the ward/hospital that the patient was admitted before this hospitalization in ICU), prior surgery, previous ICU stay, recent invasive procedures, antibiotic administration 90 days prior to KP isolation, and microbiological information (specimen types and monitoring time, the antibiotic susceptibility results). The primary outcomes of this study were 14-day all-cause mortality and 28-day all-cause mortality.

### Definitions

In our hospital, *K. pneumoniae* isolates were tested for their susceptibility to carbapenems (ertapenem, imipenem, or meropenem) and other antimicrobials by bioMerieux VITEK-2 (bioMerieux) (23). Susceptibility testing was interpreted according to the Clinical and Laboratory Standards Institute (CLSI) guidelines. *K. pneumoniae* isolates that tested intermediately or resistant to one or more carbapenems (ertapenem, imipenem, or meropenem) were considered carbapenem-resistant (24). BSI was assessed by following the criteria proposed by the US Centers for Disease Control and Prevention (25). CRKP BSI was defined by the presence of at least one CRKP-positive blood culture and symptomatic disease. CRKP non-BSIs were patients who had CRKP infections in other sites, and tested negative for CRKP in blood culture. In this study, CRKP non-bloodstream infections included CRKP respiratory infections, intra-abdominal infections, skin and soft tissue infections, urinary tract infections, and central nervous system infections. CRKP colonization was designated by the standard definition (26). In this study, mortality was considered death occurring during hospital admission, and we evaluated the time from CRKP being first isolated in the ICU to all-cause in-hospital mortality, censored at 14 and 28 days.

### Data analyses

The data were analyzed with SPSS software (version 22.0, SPSS Inc., IL, United States). Continuous variables were compared by a 2-sided t-test, and categorical variables were compared using Pearson’s Chi-square (χ^2^). Univariate and multivariate logistic regressions were performed to determine the potential risk factors for CRKP BSIs, with variables selected based on previous studies and professional experience. To identify the independent risk factors for 28-day all-cause mortality, variables with a *p* value < 0.05 in the univariate Cox regression analysis were entered into a multivariate Cox backward regression. The difference in mortality was tested using Kaplan–Meier survival analysis. A *p* value < 0.05 was considered statistically significant.

## Results

### Clinical characteristics

**Figure 1:**
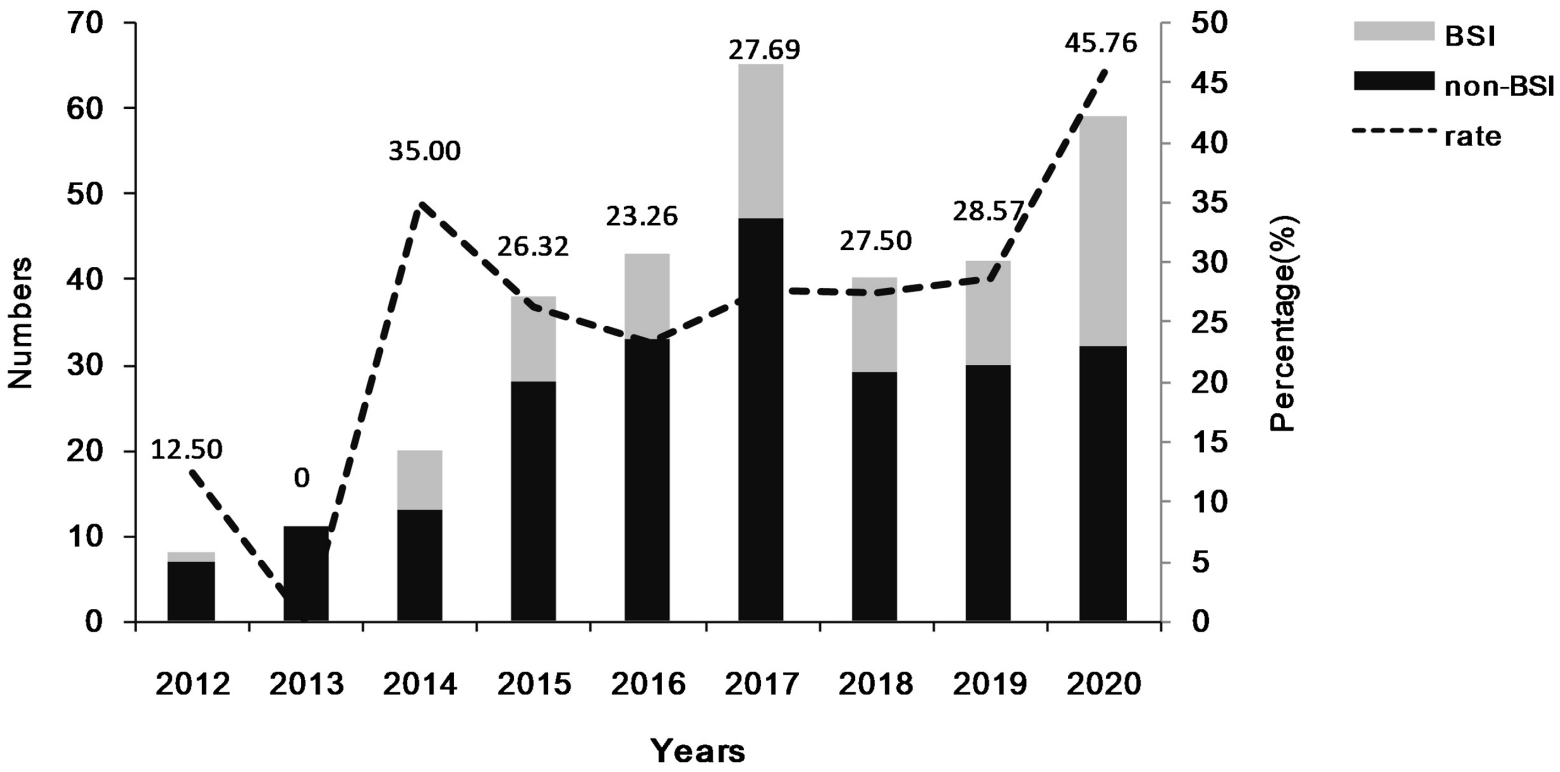
The number and percentage of patients with CRKP BSIs during the study period.

During the study period, a total of 326 patients infected with CRKP isolates were enrolled, comprising of 96 patients with CRKP BSIs and 230 patients with CRKP non-BSIs. The number and rate of patients with CRKP BSIs are presented in Figure 1. The number of patients with CRKP BSIs increased from 2012 (*n* = 1) to 2017 (*n* = 18), declined to a valley in 2018 (*n* = 11), and then increased to 2020 (*n* = 27). In general, the percentage of CRKP BSIs in CRKP infections increased from 2012 (12.50%) to 2020 (45.76%).

**Figure 2:**
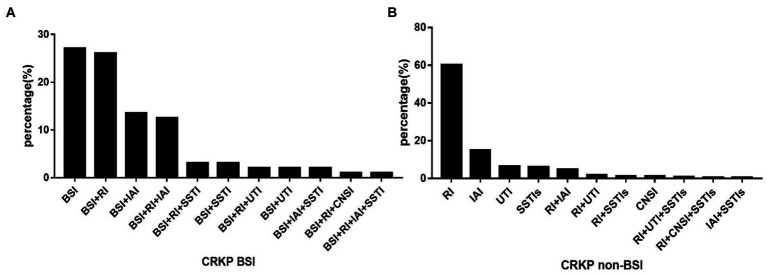
The distribution of CRKP infection types in BSI (A) and non-BSI (B) groups. BSI, bloodstream infection; RI, respiratory infection; IAI, Intra-abdominal infection; SSTIs, skin and soft tissue infections; UTI, urinary tract infection; CNSI, central nervous system infection; non-BSI, non-bloodstream infection.

The types of CRKP infections for the two groups are displayed in Figure 2. Among the patients in the CRKP BSIs group, 27.08% had CRKP BSIs only, while 72.92% had CRKP multiple organ infections. For patients who had a definite source of CRKP transmission, the respiratory tract (*n* = 25) and the abdominal cavity (*n* = 13) were the most common sources of CRKP BSIs. Other sources were the catheter (*n* = 6), skin and soft tissue (*n* = 3), and urinary tract (*n* = 2). While for the CRKP non-BSIs group, over 90% of the patients had a CRKP single organ infection (*n* = 208), with the respiratory tract (*n* = 138), abdominal cavity (*n* = 34), urinary tract (*n* = 15), and skin and soft tissue (*n* = 14) being the most common sources. Less than 10% of these patients had CRKP multiple organ infections.

### Risk factors for CRKP BSIs in CRKP-infected patients

To assess the risk factors for developing CRKP BSIs in CRKP-infected patients, we conducted a univariate logistic analysis on both BSI and non-BSI groups (Table 1). Demographic characteristics of both groups, such as sex (male), median patient age, source of patients, invasive procedures, and comorbidities, were comparable (*p* > 0.05). The results revealed that patients who had used carbapenems within 90 days were at a higher risk of acquiring BSIs (*p* = 0.016).

**Table 1:** Clinical characteristics and comparison of patients with CRKP BSIs and CRKP non-BSIs.

	All (*n* = 326)	Non-BSIs (*n* = 230)	BSIs (*n* = 96)	Univariate analysis	
				OR (95%CI)	*P*
**General characteristics**					
Sex(male)	249 (76.38)	177 (76.96)	72 (75)	0.90 (0.52–1.56)	0.705
Age(years)	54.27 ± 15.72	53.96 ± 16.42	55.02 ± 13.93	1.00 (0.99–1.02)	0.577
**Source of patients**					
Community	5 (1.53)	4 (1.74)	1 (1.04)	1.41 (0.89–2.24)	0.149
External hospitals	137 (42.02)	102 (44.35)	35 (36.46)		
Normal wards in our hospital	184 (56.44)	124 (53.91)	60 (62.50)		
**Recent events**					
Prior surgery	194 (59.51)	136 (59.13)	58 (60.42)	1.06 (0.65–1.72)	0.829
Previous ICU stay	85 (26.07)	61 (26.52)	24 (25.00)	0.92 (0.53–1.60)	0.775
**Severity of illness**					
APACHE II score	20.12 ± 7.47	19.84 ± 7.02	20.84 ± 8.40	1.02 (0.99–1.05)	0.271
SOFA	7.31 ± 3.83	7.07 ± 3.69	7.89 ± 4.09	1.06 (0.99–1.12)	0.076
**Invasive procedures**					
Tracheostomy tube	126 (38.65)	94 (40.87)	32 (33.33)	0.72 (0.44–1.19)	0.204
Surgical drainage	223 (68.40)	157 (68.26)	66(68.75)	1.02 (0.61–1.71)	0.931
Indwelled central venous catheter	193 (59.20)	133 (57.83)	60 (62.50)	1.22 (0.75–1.98)	0.434
Gastric tube	248 (76.07)	178 (77.39)	70 (72.92)	0.79 (0.46–1.36)	0.389
Urinary catheter	262 (80.37)	188 (81.74)	74 (77.08)	0.75 (0.42–1.34)	0.336
**Underlying diseases**					
Hypertension	107 (32.82)	76 (33.04)	31 (32.29)	0.97 (0.58–1.61)	0.895
Diabetes mellitus	47 (14.42)	33 (14.35)	14 (14.58)	1.02 (0.52–2.00)	0.956
Coronary	35 (10.73)	27 (11.74)	8 (8.33)	0.74 (0.32–1.70)	0.476
Hepatitis/cirrhosis	24 (7.36)	13 (5.65)	11 (11.46)	2.32 (0.95–5.67)	0.064
Malignancy	21 (6.44)	16 (6.96)	5 (5.20)	0.78 (0.28–2.22)	0.647
Cerebrovascular disease	18 (5.52)	13 (5.65)	5 (5.21)	0.92 (0.32–2.64)	0.873
**Antibiotics used within 90 days**					
Carbapenems	212 (65.03)	140 (60.87)	72 (75.00)	1.93 (1.13–3.29)	**0.016**
Tigecycline	65 (19.94)	43 (18.70)	22 (22.92)	1.29 (0.72–2.31)	0.385
Glycopeptides	93 (28.53)	69 (30)	24 (25)	0.78 (0.45–1.34)	0.363
β-lactams/β-lactamase inhibitors	242 (74.23)	177 (76.96)	65 (67.71)	0.63 (0.37–1.06)	0.083
3rd/4th generation cephalosporines	105 (32.21)	77 (33.48)	28 (29.17)	0.82 (0.49–1.37)	0.448
Fluoroquinolones	80 (24.54)	60 (26.09)	20 (20.83)	0.75 (0.42–1.32)	0.316
Aminoglycosides	22 (6.75)	17 (7.39)	5 (5.20)	0.69 (0.25–1.92)	0.476
Antifungal drugs	71 (21.78)	51 (22.17)	20 (20.83)	0.92 (0.52–1.65)	0.789

The bold value is statistically significant in multivariate logistic analysis (*P* = 0.019).

Based on the univariate analysis, we selected the factors with *p* values less than 0.05 or professional experience for the multivariate logistic analysis. The prior use of carbapenems within 90 days was identified as an independent risk factor for CRKP BSI (*p* = 0.019).

### Outcomes of CRKP BSIs and non-BSIs

**Figure 3:**
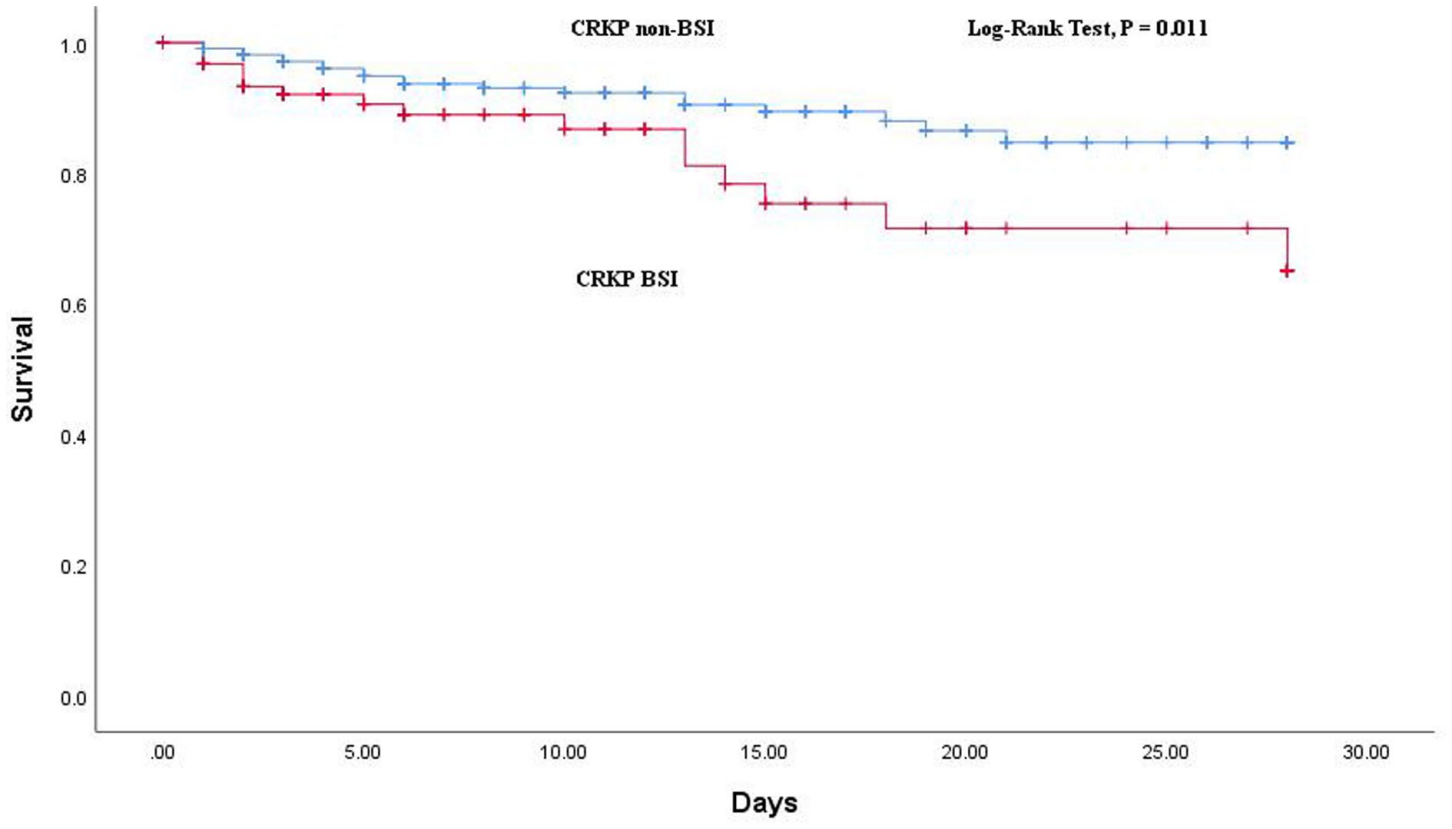
Kaplan–Meier curves showing the impact of CRKP BSI on survival at 28 days.

The comparison of outcomes between CRKP BSIs and CRKP non-BSIs is shown in Table 2. The BSI group demonstrated a higher rate of 28-day all-cause mortality (*p* = 0.036) and a tendency toward non-susceptible to more carbapenems (*p* = 0.001). Additionally, the Kaplan–Meier curve revealed a significant difference in 28-day all-cause mortality between the two groups (log-rank test: *p* = 0.011; Figure 3).

**Table 2:** Comparison of outcomes of patients with CRKP BSIs and CRKP non-BSIs.

	Non-BSIs (*n* = 230)	BSIs (*n* = 96)	χ^2^/t	*P*
Number of tested carbapenems that CRKP isolates non-susceptible against	2.61 ± 0.66	2.85 ± 0.48	−3.73	0.001
Length of ICU stay	22.66 ± 20.12	19.11 ± 15.33	1.55	0.123
14 day mortality	16 (6.96)	13 (13.54)	3.62	0.057
28 day mortality	20 (8.69)	16 (16.67)	4.38	0.036

### Risk factors of 28-day all-cause mortality in CRKP-infected patients

Univariable and multivariable analyses of risk factors associated with 28-day all-cause mortality in CRKP-infected patients are shown in Table 3. The variables that showed statistically significant differences (*P* < 0.05) in the univariate Cox regression analysis were included in a multivariate Cox backward regression. The analysis revealed that a high SOFA score (*p* = 0.000), CRKP isolates non-susceptible against more tested carbapenems (*p* = 0.025), and the use of antifungal drugs within the prior 90 days (*p* = 0.018) were significant factors for 28-day all-cause mortality in CRKP-infected patients.

**Table 3:** Cox proportional hazards regression analysis of predictors associated with 28-day mortality in CRKP-infected patients.

	Univariate analysis		Multivariate analysis	
	HR (95%CI)	*P*	HR (95%CI)	*P*
Sex (male)	0.67 (0.32–1.38)	0.273		
Age (years)	1.01 (0.99–1.03)	0.451		
Source of patients	1.02 (0.56–1.87)	0.939		
**CRKP infections**				
CRKP BSI	2.28 (1.18–4.41)	0.014		
CRKP multiple organ infections	1.29 (0.67–2.51)	0.450		
Number of tested carbapenems that CRKP isolates non-susceptible against	4.79 (1.34–17.15)	0.016	4.41 (1.21–16.06)	0.025
Septic shock/sepsis at ICU admission	2.09 (1.08–4.04)	0.028		
**Severity of illness**				
APACHE II score	1.09 (1.05–1.13)	0.000		
SOFA	1.19 (1.11–1.29)	0.000	1.16 (1.08–1.25)	0.000
**Antibiotics used within 90 days**				
Carbapenems	3.33 (1.38–8.01)	0.007		
Tigecycline	1.45 (0.70–3.01)	0.318		
Glycopeptides	0.74 (0.34–1.63)	0.455		
β-lactams/β-lactamase inhibitors	1.19 (0.54–2.61)	0.674		
3rd/4th generation cephalosporines	0.50 (0.22–1.14)	0.101		
Fluoroquinolones	1.37 (0.66–2.84)	0.402		
Aminoglycosides	1.19 (0.37–3.89)	0.770		
Antifungal drugs	2.23 (1.35–3.68)	0.002	1.84 (1.11–3.03)	0.018
Length of ICU stay	0.98 (0.96–1.00)	0.054		

## Discussion

In our study, we reported on the epidemiology, risk factors, and outcomes of CRKP BSIs and CRKP non-BSIs. Notably, we focused on patients infected with CRKP and identified the increasing prevalence of CRKP BSI. We found that the risk factors for CRKP BSI were present in CRKP-infected patients, while previous reports have focused on KP patients or non-CRKP infected patients. To our knowledge, this is the first study to identify the risk factors and outcomes of CRKP BSIs through comparison to CRKP non-BSIs.

Previous studies have shown that the rate of CRKP BSI in KP BSI has increased throughout the study (27, 28). And our study has revealed that the proportion of CRKP BSI in CRKP infections has progressively increased over the study period. These all can reflect an increase in the incidence rate of CRKP BSI. Among patients with positive CRKP blood cultures, 27.08% had no identified source of CRKP infection. This could be due to either a primary infection or secondary infection with an unknown source. In addition, 72.92% of patients with CRKP BSIs had other sites of CRKP infection. Furthermore, it was observed that the percentage of patients with a single episode of CRKP infection in the non-BSI group exceeded 90%. The most commonly identified CRKP non-bloodstream samples were respiratory tract samples. It is worth noting that the distribution of CRKP in clinical samples varied by region (29). By exploring CRKP’s local distribution, we can help control its diffusion and transmission.

In our study, we have identified the prior use of carbapenems within 90 days as an independent risk factor for CRKP BSIs. It is possible that the production of carbapenemase and porin deficiency contribute to carbapenem resistance in *K. pneumoniae* during carbapenem therapy (30). And clinical retrospective analysis has revealed that previous exposure to carbapenems has been proven to have a significant correlation with the acquisition of CRKP compared to CSKP (17). In addition, the annual rate of CRKP in KP BSI has increased over the study period. Therefore, we infer that the prior use of carbapenems is a understandable risk factor for CRKP BSIs. However, for patients with *Acinetobacter* spp. bloodstream infections, the rate of prior use of carbapenems was not significantly higher than those who were non-BSI patients of *Acinetobacter* spp. (19). As few studies have identified risk factors for CRKP BSIs in comparison to CRKP non-BSIs, further research is necessary to validate this finding. Although the CRKP BSI group had a tendency to have more male and older patients than the non-BSI group, we found no statistically significant differences. It has been observed that patients with bacteremic pneumococcal pneumonia are significantly younger than non-bacteremic patients (20, 31). Since there is a lack of relevant data on this topic, comparisons between studies of different microbes and populations are necessary.

Patients with CRKP BSI had multiple organ infections with CRKP, indicating the complexity of CRKP dissemination in BSI. The CRKP isolates in the BSI group tended to be not susceptible to more carbapenems, which made treatment more difficult, leading to a significant association with poor clinical outcomes. Furthermore, the BSI group had a higher incidence of 28-day all-cause mortality. Previous studies with different control groups (15, 18) have also reported similar poor clinical outcomes in the CRKP BSI group.

In the study, data on antimicrobial susceptibility testing were collected. We analyzed on the resistance of each KP strain against ertapenem, imipenem, and meropenem, as well as the number of tested carbapenems that CRKP isolates were non-susceptible to. The non-susceptibility of CRKP isolates to more tested carbapenems, a high SOFA score, and prior antifungal use are independent risk factors for 28-day all-cause mortality in CRKP BSI patients. As anticipated, previous studies have also determined that a high SOFA score increases mortality risk in critically ill patients with carbapenem-resistant bacteria infections (32, 33). For the first time, this study demonstrates that prior antifungal use is an independent predictor of mortality in adult patients with CRKP infections. Previous studies have reported this trend in neonatal patients (34). Additionally, few studies have reported the severity of carbapenem non-susceptibility in CRKP isolates, and some of our findings are novel and warrant further analysis. Further exploration in other medical centers is needed, and it may help to stratify ICU patients based on their local risk of mortality.

Our study had some limitations. Firstly, this is a retrospective observational analysis conducted at a single center, which makes it prone to selection bias. Secondly, the number of respiratory samples collected was significantly higher than that of other specimen types, which may be attributed to the easier sampling. In contrast, blood collection is more discreet. Finally, in-hospital mortality was a widely measured outcome in both China and other countries. In-hospital mortality was lower than crude mortality in China, which may limit its external validity in other countries. Nevertheless, given the large time span of our study, the available data were carefully reviewed.

In summary, the proportion of CRKP BSI increased progressively in CRKP-infected patients. The use of carbapenems within the prior 90 days was an independent risk factor for CRKP BSIs. The non-susceptibility of CRKP isolates to more tested carbapenems and a higher mortality rate were found in the CRKP BSI group. Our findings may assist physicians in recognizing CRKP BSI sooner and stratifying ICU patients by risk of mortality to initiate the appropriate therapeutic strategies.

## Data availability statement

The original contributions presented in the study are included in the article/supplementary material, further inquiries can be directed to the corresponding author.

## Ethics statement

The studies involving humans were approved by the Ethics Committee of Xiangya Hospital Central South University. The studies were conducted in accordance with the local legislation and institutional requirements. Written informed consent for participation was not required from the participants or the participants’ legal guardians/next of kin in accordance with the national legislation and institutional requirements.

## Author contributions

PW and TY contributed to the conception and design of the study. XZ managed the data collection. PW and BZ analyzed the data. PW wrote the initial draft. XS submitted and revised the article. All authors contributed to the article and approved the submitted version.

## Conflict of interest

The authors declare that the research was conducted in the absence of any commercial or financial relationships that could be construed as a potential conflict of interest.

## Publisher’s note

All claims expressed in this article are solely those of the authors and do not necessarily represent those of their affiliated organizations, or those of the publisher, the editors and the reviewers. Any product that may be evaluated in this article, or claim that may be made by its manufacturer, is not guaranteed or endorsed by the publisher.
